# Insufficient impact of the aldose reductase inhibitor cemtirestat on the skeletal system in type 2 diabetic rat model

**DOI:** 10.1371/journal.pone.0336508

**Published:** 2025-11-10

**Authors:** Monika Martiniakova, Marta Soltesova Prnova, Veronika Kovacova, Vladimira Mondockova, Karol Svik, Piotr Londzin, Joanna Folwarczna, Radoslav Omelka

**Affiliations:** 1 Department of Zoology and Anthropology, Faculty of Natural Sciences and Informatics, Constantine the Philosopher University in Nitra, Nitra, Slovakia; 2 Centre of Experimental Medicine, Institute of Experimental Pharmacology and Toxicology, Slovak Academy of Sciences, Bratislava, Slovakia; 3 Faculty of Informatics and Information Technologies, Slovak University of Technology in Bratislava, Bratislava, Slovakia; 4 Department of Botany and Genetics, Faculty of Natural Sciences and Informatics, Constantine the Philosopher University in Nitra, Nitra, Slovakia; 5 Department of Pharmacology, Faculty of Pharmaceutical Sciences in Sosnowiec, Medical University of Silesia in Katowice, Sosnowiec, Poland; Istanbul Health and Technology University, Faculty of Medicine, TÜRKIYE

## Abstract

Cemtirestat, a multi-target drug combining aldose reductase inhibition with antioxidant properties, is considered a promising therapeutic agent for chronic diabetic complications. Current evidence suggests that long-standing diabetes adversely affects skeletal health, leading to diabetic bone disease. As the impact of cemtirestat on the skeletal system in an animal model of type 2 diabetes mellitus (T2DM) is still unknown, our study first investigated its effect on impaired bone health in Zucker diabetic fatty (ZDF) rats. Adult rats were divided into four groups: L (untreated lean ZDF rats), D (untreated obese ZDF rats), DT2.5 (obese ZDF rats treated with 2.5 mg/kg/day cemtirestat), and DT7.5 (obese ZDF rats treated with 7.5 mg/kg/day cemtirestat), with cemtirestat treatment lasting 2 months. Group D had increased levels of plasma glucose, insulin, triglycerides, glycated hemoglobin, total cholesterol, alkaline phosphatase, alanine aminotransferase, C-terminal telopeptide of type 1 collagen, greater body weight, femoral weight, structure model index, reduced cortical bone volume fraction, cortical bone thickness, trabecular bone volume fraction, and trabecular thickness compared to group L. Cemtirestat supplementation only elevated plasma phosphate levels in group DT2.5, trabecular bone volume fraction and trabecular thickness in group DT7.5, but the treatment had no effect on other parameters demonstrated in ZDF rats by macroscopic analysis, micro-CT cortical bone analysis, and mechanical testing. These findings indicate that the efficacy of cemtirestat in restoring deteriorated bone health caused by T2DM is not substantiated due to its insufficient effect on the skeletal system in the ZDF rat model.

## Introduction

With increasing age, alterations in lifestyle habits, and changed dietary patterns in humans, diabetes mellitus (DM) has become the third most dangerous non-communicable disease after cardiovascular disorders and cancer [1]. The most widespread form of the disease is type 2 diabetes mellitus (T2DM), which is characterized by chronic hyperglycemia, insulin resistance, and ineffective insulin secretion and action. Globally, the prevalence of T2DM has raised dramatically in recent decades, placing a significant burden on patients, their families, and the healthcare system. By 2035, 592 million individuals worldwide are expected to experience T2DM [2–5]. In general, chronic hyperglycemia in T2DM is associated with the generation of reactive oxygen species (ROS), increased accumulation of advanced glycation end products (AGEs), which suppress osteoblast activity, induce osteocyte apoptosis, increase collagen cross-linking, and a pro-inflammatory senescence phenotype. Together with a higher incidence of microvascular disease and an elevated risk of vitamin D deficiency, the aforementioned factors significantly contribute to the deterioration of bone quality and health [6–9].

The skeleton is increasingly being recognized as a site of end-organ damage in DM. The effects of DM on the skeletal system are complex and under active investigation. Diabetic bone disease is commonly manifested by low bone turnover, disturbed bone microarchitecture, impaired bone mechanical properties, delayed bone regeneration, and is considered secondary osteoporosis induced by DM [1,7,10,11]. Numerous studies have shown that despite higher and/or unaltered bone mineral density (BMD), the overall risk of fracture is greater in individuals with T2DM, being higher with poor glycemic control, longer disease duration, and diabetic complications [12,13]. Overall, older patients with T2DM were found to be more susceptible to fractures of the hip, foot, humerus, upper leg, and total fractures. Additionally, post-fracture subjects were more likely to have falls, prolonged fracture healing, and elevated mortality [3,14–18].

In general, the polyol pathway serves as the main link responsible for glucose toxicity in DM [19]. This pathway catalyzes the conversion of excess glucose to sorbitol at the expense of nicotinamide adenine dinucleotide phosphate (NADPH) by the enzyme aldose reductase (AR) and subsequently to fructose at the expense of nicotinamide adenine dinucleotide (NAD+), leading to the production of nicotinamide adenine dinucleotide (hydrogenated) (NADH) by the enzyme sorbitol dehydrogenase. All consequences of the polyol pathway, including NADPH consumption, sorbitol accumulation, fructose and NADH production, are linked to the pathogenesis of DM and its complications. They contribute significantly to the formation of glycolytic intermediates and AGEs, thereby increasing oxidative stress, osmotic stress, and glycotoxicity, which can lead to inflammation and growth factor imbalance [20,21]. Current research points to a primary role of AR in the development of chronic diabetic complications. AR is therefore considered a crucial therapeutic target [22,23]. It has been demonstrated that AR is present in the periosteum, endosteum, osteocytes, and endothelial layer of blood vessels in rat bone [24] and that the polyol pathway may be closely involved in the development of altered bone metabolism [25]. Several AR inhibitors (ARIs) have been identified. They are particularly used to manage DM-related complications and their mode of action is to reduce the conversion of glucose into sorbitol within cells [26–29]. Cemtirestat (3-mercapto-5H-1,2,4-triazino [5,6-b] indole-5-acetic acid) has been developed as a multi-target drug combining AR inhibition with antioxidant and ROS scavenging properties [30,31]. In experimental rat models of DM, cemtirestat has demonstrated noticeable neuroprotective effects. In streptozotocin (STZ)-induced rat model of type 1 DM (T1DM), cemtirestat attenuated the symptoms of peripheral neuropathy, improved indices of oxidative stress, reduced hypertriglyceridemia, and inhibited sorbitol accumulation in erythrocytes [23]. In Zucker diabetic fatty (ZDF) rat model of T2DM, cemtirestat normalized symptoms of peripheral neuropathy, partially inhibited sorbitol accumulation in the sciatic nerve and erythrocytes [32], and reduced lipid peroxidation in the brain cortical slices [30]. Considering cemtirestat impact on the skeletal system in DM, our previous study revealed inadequate effects of its long-term treatment on impaired bone health in STZ-induced diabetic rats [33]. However, the impact of cemtirestat on the skeletal system in an animal model of T2DM is still unclear. Therefore, our study aimed to first investigate whether cemtirestat treatment is able to improve deteriorated bone quality in ZDF rats.

## Materials and methods

### Animals and procedures

Male ZDF rats (obese diabetic fa/fa) and control lean rats from the same litter (fa/+) were bred at the Department of Toxicology and Laboratory Animal Breeding, Slovak Academy of Sciences (SAS), Dobra Voda. Relative humidity (40–70%), photoperiod (12:12 light:dark cycle), and temperature (22–24 °C) were all controlled and monitored. Rats had unrestricted access to tap water and standard rodent chow (19.2% protein, 65.1% carbohydrate, 4.0% fat, 4.0% fiber, 7.7% ash by weight) throughout the experiment. At the end of the 5th month, rats were divided into four groups of six animals each: L (untreated lean ZDF rats), D (untreated obese ZDF rats), DT2.5 (obese ZDF rats treated with cemtirestat at a dose of 2.5 mg/kg/day), and DT7.5 (obese ZDF rats treated with cemtirestat at a dose of 7.5 mg/kg/day). Cemtirestat was administered to experimental animals by oral gavage as an aqueous solution in tap drinking water during 2 months of drug therapy. Doses of cemtirestat were chosen based on published studies [21,32]. Untreated rats received only water during the same period. The study was approved by the Ethics Committee of the Department of Toxicology and Laboratory Animal Breeding, SAS, and the State Veterinary and Food Administration of the Slovak Republic (No. 335/18–221).

### Biochemical analysis

At the end of the experiment, the blood was collected by cardiac puncture under total anaesthesia by Zoletil^®^/Xylazine mixture (100 mg/kg Zoletil^®^ and 16 mg/kg Xylazine). The following parameters were measured (Laboratoria s.r.o., Piestany, Slovakia): glucose, insulin, triglycerides, total cholesterol, urea, creatinine, alkaline phosphatase (ALP), alanine aminotransferase (ALT), aspartate aminotrasferase (AST), gamma-glutamyl transferase (GGT), calcium (Ca), phosphate (P), and magnesium (Mg). Glycated hemoglobin was determined by the rat HbA1c kit of Crystal Chem Inc (Elk Grove Village, IL, USA). Bone turnover markers – procollagen type I N-propeptide (P1NP, a marker of bone formation) and C-terminal telopeptide of type 1 collagen (CTX, a marker of bone resorption) were measured using commercial assay rat PINP ELISA kit (FineTest, Wuhan, China) and rat beta Crosslaps ELISA kit (Antibodies, Taipei, Taiwan), respectively.

### Macroscopic analysis

Rats from each group had their body weight measured at the end of the experiment. Macroscopic parameters (femoral weight, femoral length) were determined using both femurs (n = 48).

### Microcomputed tomography (micro-CT)

Cortical and trabecular bone microstructure were assessed using micro-CT (μCT 50, Scanco Medical, Brüttisellen, Switzerland). Femoral bones were stored at −18°C and wrapped in PBS-soaked gauze prior to analysis. High resolution scans with a voxel size of 14.8 μm were obtained. Because of significantly lower femoral lenght in diabetic rats, the region of interest for cortical bone analysis was selected at a distance of 11.30 mm from the end of the growth plate (EGP), whereas that for lean rats was selected at a distance of 12.02 mm from the EGP and extending 2 mm at the femoral midshaft. Trabecular bone was scanned at a distance of 2.61 mm from the EGP for diabetic rats, whereas that for lean ones was scanned at a distance of 2.77 mm from the EGP and extending 2 mm. Scanning parameters comprised 0.5 mm aluminum filter, 200 mA current, 300 ms integration time, and 70 kVp voltage. The Scanco microCT Evaluation Program V6.6 was used for quantitative analysis. Cortical metrics included cortical bone volume fraction (BV/TV), volumetric cortical bone mineral density (BMD), cortical bone thickness, bone surface, bone area, polar moment of inertia (pMOI), and maximum (Imax/Cmax) and minimum (Imin/Cmin) loading resistance. Trabecular measurements involved trabecular bone volume fraction (BV/TV), volumetric trabecular bone mineral density (BMD), trabecular number, thickness, separation, bone surface, connectivity density, and structure model index.

### Mechanical testing

Using an Instron 3342 500 N apparatus (Instron, Norwood, MA, USA), mechanical properties of the femoral diaphysis were assessed using a three-point bending test. Data analysis was conducted using Bluehill 2 version 2.14 software (Instron, Norwood, MA, USA). The femur was placed on two supporting points spaced 16 mm apart. The load was applied perpendicular to the bone’s long axis, in the middle of the femoral length. Following preconditioning to ensure stable bone positioning, a proper test was initiated with a 100 Hz sampling rate and a 0.01 mm/s displacement rate. The load, displacement, and energy for the yield point (0.05% offset) and maximum load point were evaluated as extrinsic parameters. Additionally, the stress values for the yield point and maximum load point (intrinsic parameters) were calculated. In order to assess the moment of inertia in the break-section, it was assumed that the femoral diaphysis was a circular beam. A digital caliper (Topex, Warsaw, Poland) was used to measure the mean diameter of the femoral diaphysis at the mid-length of the bone.

### Statistical evaluation

Statistical evaluation was conducted using IBM SPSS Statistics 26.0 software (New York, NY, USA). The data were presented as mean ± standard deviation (SD). ANOVA combined with post hoc tests (Tukey’s and/or Games-Howell) was used to detect differences in all parameters analyzed. The normal distribution of data was checked using the Shapiro-Wilk test, while the homogeneity of variance was tested using the Levene’s test. Statistically significant p value was defined as less than 0.05.

## Results

### Biochemical analysis

**Fig 1:**
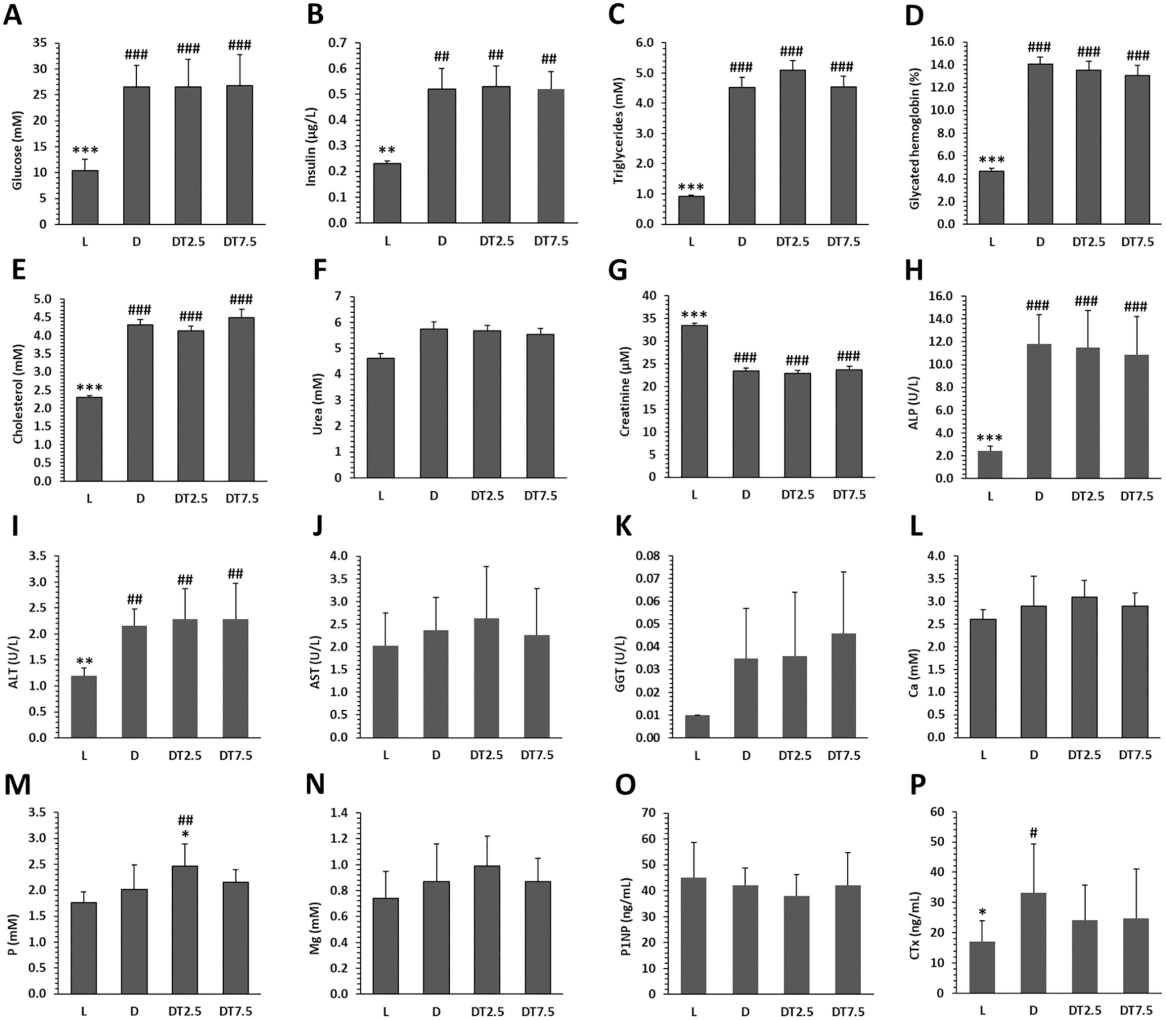
Biochemical markers investigated in lean ZDF rats (L), obese ZDF rats (D), and those treated with cemtirestat at doses of 2.5 mg/kg/day (DT2.5), and 7.5 mg/kg/day (DT7.5) for 2 months. (A) Plasma glucose, (B) insulin, (C) triglycerides, (D) glycated hemoglobin, (E) total cholesterol, (F) urea, (G) creatinine, (H) alkaline phosphatase (ALP), (I) alanine aminotransferase (ALT), (J) aspartate aminotrasferase (AST), (K) gamma-glutamyl transferase (GGT), (L) calcium (Ca), (M) phosphate (P), (N) magnesium (Mg), (O) procollagen type I N-propeptide (P1NP), (P) C-terminal telopeptide of type 1 collagen (CTX). Significant differences compared to group D: p < 0.05 (*), p < 0.01 (**), and p < 0.001 (***). # Significant changes in relation to group L: p < 0.05 (#), p < 0.01 (##), and p < 0.001 (###).

During the entire experiment, persistent hyperglycemia above 25 mmol/l was recorded in groups D, DT2.5, and DT7.5. Glycated hemoglobin levels above 13% indicated severe hyperglycemia in these groups. Concentrations of plasma glucose, insulin, triglycerides, glycated hemoglobin, total cholesterol, ALP, ALT, and CTX in group D were significantly higher than in group L. Conversely, increased creatinine levels were observed in group L. Plasma urea, AST, GGT, Ca, P, Mg, and P1NP levels were not influenced by T2DM. In group DT2.5, only plasma P levels were elevated compared to group D; other biochemical markers were unaffected by cemtirestat administration to diabetic rats (Fig 1, S1 Table).

### Macroscopic analysis

**Fig 2:**
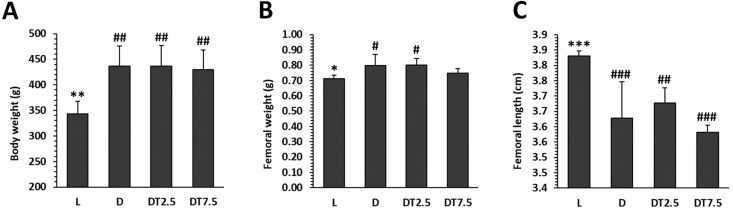
Macroscopic parameters investigated in lean ZDF rats (L), obese ZDF rats (D), and those treated with cemtirestat at doses of 2.5 mg/kg/day (DT2.5), and 7.5 mg/kg/day (DT7.5) for 2 months. (A) Total body weight, (B) femoral weight, (C) femoral length. Significant differences compared to group D: p < 0.05 (*), p < 0.01 (**), and p < 0.001 (***). # Significant changes in relation to group L: p < 0.05 (#), p < 0.01 (##), and p < 0.001 (###).

The body weight and femoral weight were significantly greater in group D compared to group L. On the contrary, femurs had a longer length in group L. After treatment with cemtirestat, no significant differences were found in the mentioned parameters between groups D and DT2.5, as well as D and DT7.5. The findings are shown in Fig 2 and S1 Table.

### Microcomputed tomography (micro-CT)

**Fig 3:**
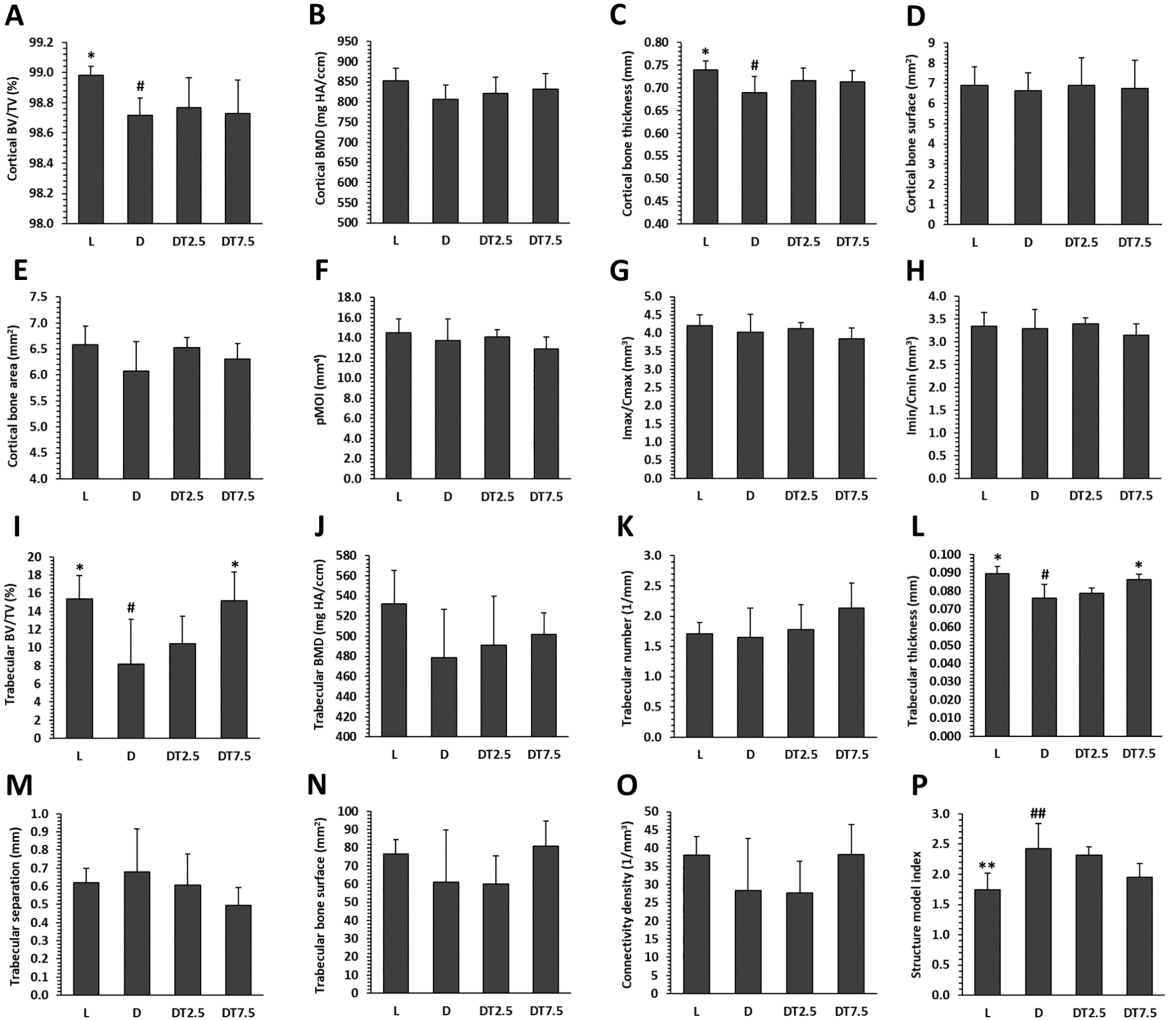
Micro-CT cortical and trabecular bone metrics of the femur in lean ZDF rats (L), obese ZDF rats (D), and those treated with cemtirestat at doses of 2.5 mg/kg/day (DT2.5), and 7.5 mg/kg/day (DT7.5) for 2 months. (A) cortical bone volume fraction (BV/TV), (B) volumetric cortical bone mineral density (BMD), (C) cortical bone thickness, (D) cortical bone surface, (E) cortical bone area, (F) polar moment of inertia (pMOI), (G) maximum loading resistance (Imax/Cmax), (H) minimum loading resistance (Imin/Cmin), (I) trabecular bone volume fraction (BV/TV), (J) volumetric trabecular bone mineral density (BMD), (K) trabecular number, (L) trabecular thickness, (M) trabecular separation, (N) trabecular bone surface, (O) connectivity density (P) structure model index. Significant differences compared to group D: p < 0.05 (*), p < 0.01 (**). # Significant changes in relation to group L: p < 0.05 (#), p < 0.01 (##).

**Fig 4:**
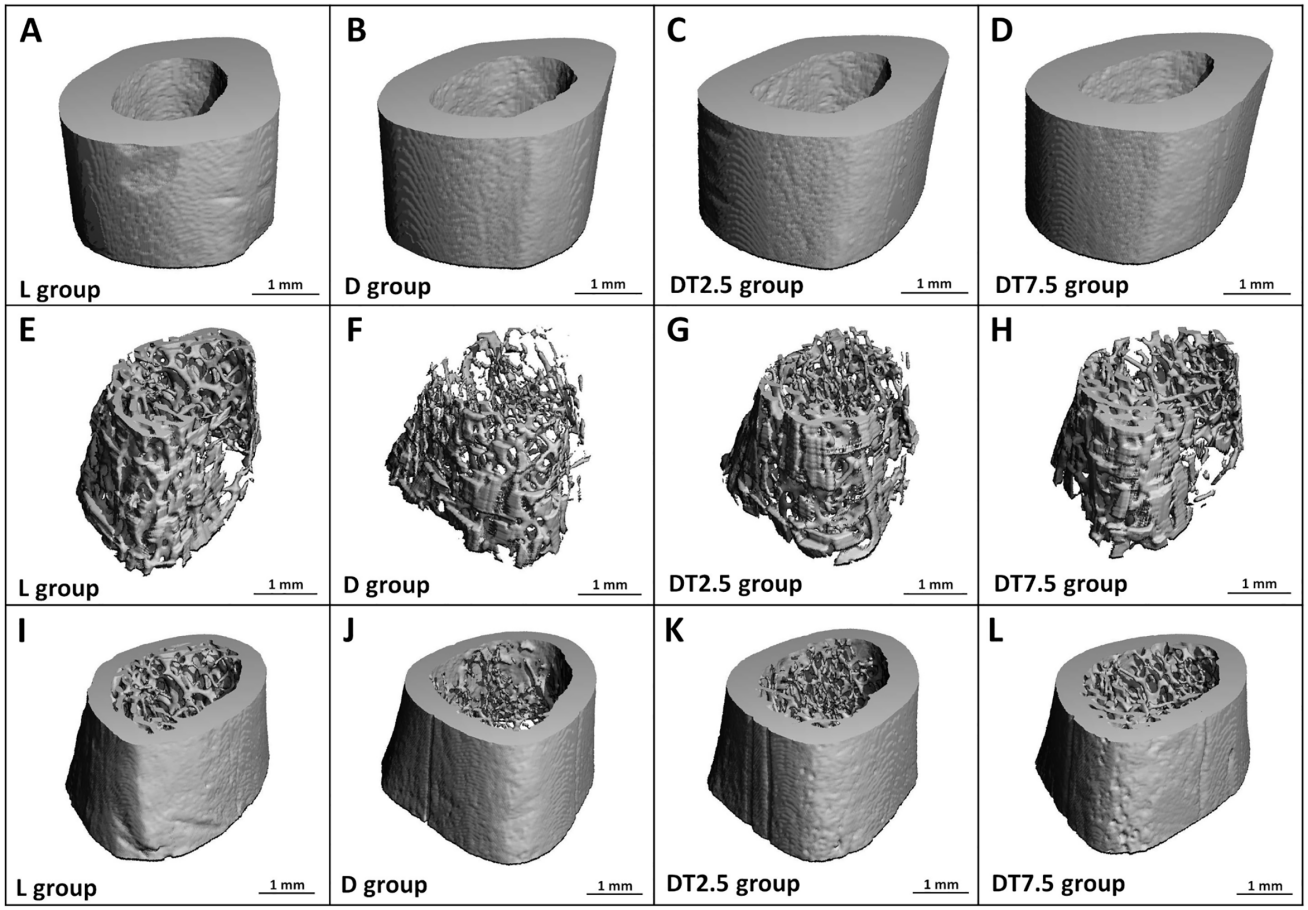
Representative micro-CT images of cortical bone in femoral diaphysis (A-D), trabecular bone in distal metaphysis (E-H), and both bone compartments in distal metaphysis (I-L).

In group D, a significant decrease in cortical BV/TV, cortical bone thickness, trabecular BV/TV, trabecular thickness, and an increase in structure model index were determined compared to group L. Volumetric cortical BMD, bone surface, bone area, pMOI, Imax/Cmax, Imin/Cmin, volumetric trabecular BMD, trabecular number, separation, bone surface, and connectivity density were unaffected by T2DM (Fig 3, S1 Table). Cemtirestat treatment had no apparent effect on any of the cortical bone metrics measured in diabetic rats. In trabecular bone, BV/TV and trabecular thickness were found to be significantly higher in group DT7.5 than in group D. Fig 4 displays representative micro-CT images of cortical and trabecular bone in each experimental group.

### Mechanical testing

**Fig 5:**
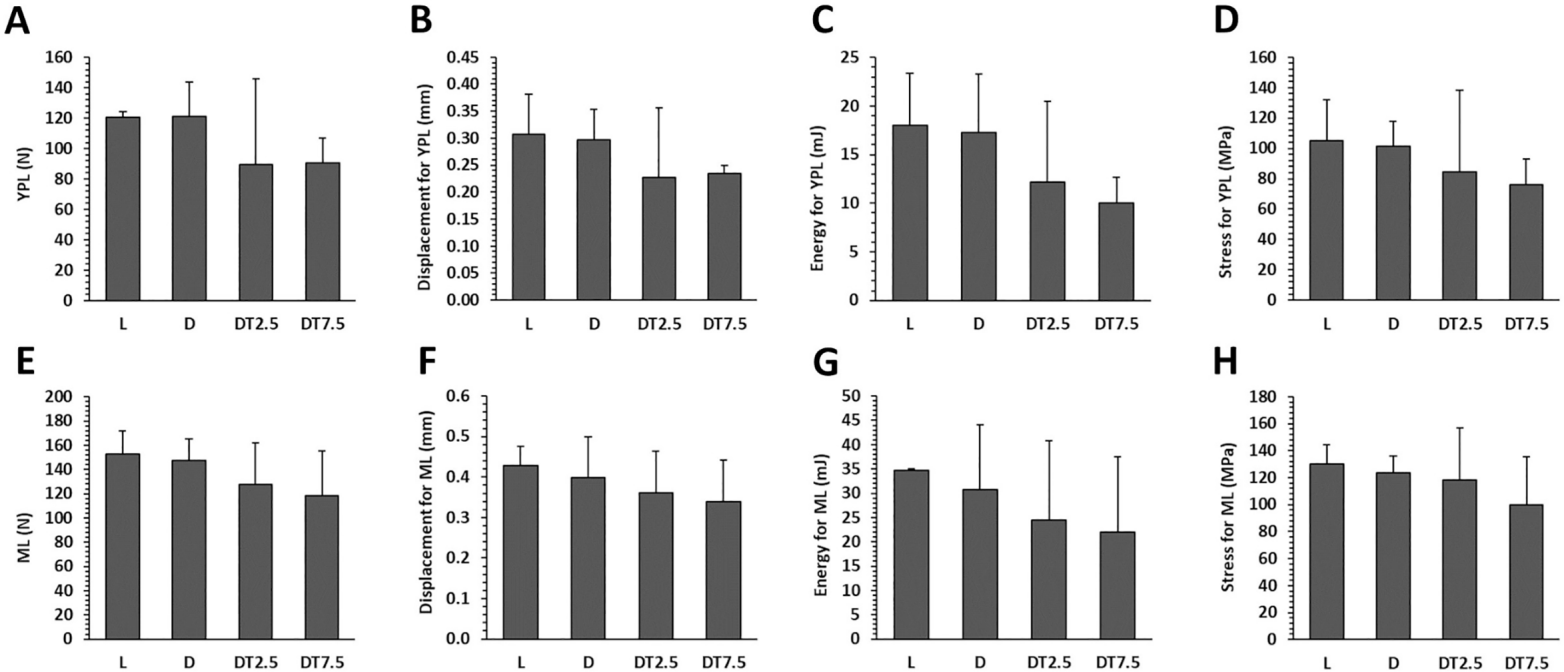
Mechanical properties of femoral bones in lean ZDF rats (L), obese ZDF rats (D), and those treated with cemtirestat at doses of 2.5 mg/kg/day (DT2.5), and 7.5 mg/kg/day (DT7.5) for 2 months. (A) yield point load (YPL), (B) displacement for yield point load, (C) energy for yield point load, (D) stress for yield point load, (E) maximum load, (F) displacement for maximum load, (G) energy for maximum load, (H) stress for maximum load. Significant differences compared to group D: p < 0.05 (*). # Significant changes in relation to group L: p < 0.05 (#).

Although almost all parameters related to mechanical properties of the femoral diaphysis were slightly, but insignificantly higher in group L, no significant differences were detected between groups D and L. In diabetic rats, cemtirestat administration had no noticeable effect on all extrinsic and intrinsic parameters examined (Fig 5, S1 Table).

## Discussion

Male ZDF rats were used as a suitable animal model of T2DM to monitor diabetic complications linked to the skeletal system after cemtirestat treatment in our study. Leptin receptor deficiency is a common feature of the ZDF rat model, which manifests with functional characteristics of T2DM such as hyperphagia, severe hyperglycemia, hyperlipidemia, hyperinsulinemia in males at 9–10 weeks of age. These rats suffer from metabolic syndrome with obesity and insulin resistance, similar to humans. Additionally, they exhibit reduced bone mass, lower bone strength, and delayed bone regeneration [10,34–36]. Rats from group D showed significantly greater levels of plasma glucose, insulin, glycated hemoglobin, triglycerides, total cholesterol, ALP, ALT, and CTX than those from group L, indicating that this model is sufficient overall. Significantly higher concentrations of aforementioned biochemical parameters were also determined in ZDF rats in other studies [7,32,37–43]. Considering bone turnover markers, P1PN levels were insignificantly lower and CTX levels were demonstrably higher in group D versus group L. Identically, Cheng et al. [44] found non-significantly decreased P1NP levels but considerably increased CTX levels when comparing diabetic and non-diabetic ZDF rats. A lower rate of bone formation, manifested by significantly reduced P1NP levels and a higher rate of bone resorption, followed by increased CTX levels in ZDF rats, was documented by Picke et al. [10]. Similar associations as stated in the previous study were also identified by Kubota et al. [39] but with different markers of bone turnover (osteocalcin – a marker of bone formation, tartrate-resistant acid phosphatase 5b - a marker of bone resorption) in ZDF rats. Fajardo et al. [45], however, reported significantly reduced values of both P1NP and CTX in the ZDF rat model. In our study, T2DM did not influence noticeably plasma levels of urea, AST, GGT, Ca, P, and Mg. On the contrary, some investigations reported slightly higher concentrations of urea, AST, Ca, P in a rat model of T2DM [10,39]. Consistent with our findings, Wang et al. [40] revealed non-significant rises in blood Ca and P levels in obese compared to lean ZDF rats. In our study, 2-month cemtirestat therapy significantly increased only plasma P levels in group DT2.5, other biochemical markers were not affected in groups DT2.5 and DT7.5. In general, P is an essential component of hydroxyapatite crystals in bone and its chronic deficiency leads to impaired skeletal mineralization [46]. In the blood, P level is influenced by age, diet, and pH [47]. Homeostasis of P is achieved through complex interactions between bone and multiple organs (e.g., kidney, intestine, parathyroid glands) via numerous hormones including parathyroid hormone, calcitriol, fibroblast growth factor 23, and 1,25-dihydroxyvitamin D3 [48,49]. Disturbances in P homeostasis can therefore impair the functions of many organ systems. Previous experiments revealed that cemtirestat treatment for 4 months demonstrably reduced plasma triglyceride levels in STZ-induced diabetic rat model [23,33]. For this reason, there is an assumption that a longer period of therapy might have a substantial impact on the lipid profile even in ZDF rats.

Rats from group D showed higher body weight, femoral weight but shorter femoral length than those from group L, indicating a small deficit in longitudinal growth despite greater total body weight, in agreement with our earlier findings [7]. It is widely recognized that T2DM adversely affects bone size. Smaller femoral length has also been determined in obese versus lean ZDF rats in multiple studies [37,50–52]. Nevertheless, in diabetic rats, cemtirestat therapy was unable to reverse this bone alteration.

Disrupted skeletal microarchitecture, evidenced by significantly decreased metrics of cortical and trabecular bone compartments in group D (cortical BV/TV, cortical bone thickness, trabecular BV/TV, trabecular thickness, structure model index), points to microstructural abnormalities associated with T2DM, which were also supported by previous research [7,10,37,39,41,44,50,53,54]. In our study, T2DM did not influence certain femoral bone parameters determined by micro-CT. In this context, Cheng et al. [44] and Reinwald et al. [50] found no obvious changes in trabecular number between diabetic and non-diabetic ZDF rats. According to Zeitoun et al. [53], obese ZDF rats had non-significantly lower volumetric cortical BMD, cortical perimeter, and connectivity density versus their lean controls. Volumetric cortical BMD was not affected by T2DM either in the study by Kubota et al. [39]. Our results subsequently showed that cemtirestat treatment in diabetic rats had no discernible impact on any of the cortical bone quantities. On the other hand, compared to group D, rats from group DT7.5 had considerably greater trabecular BV/TV, which was due to thicker trabeculae and not their increased number. This demonstrates the positive influence of cemtirestat on aforementioned parameters. Several studies have reported a differential effect of certain drugs on cortical versus trabecular bone in ZDF rats [7,10]. Trabecular bone undergoes faster bone turnover because it has a large surface area exposed to bone marrow and blood flow. Therefore, in comparison with cortical bone, the impact of drugs can be manifested faster by changing its microarchitecture. In a rat model of T1DM, cemtirestat supplementation for 4 months did not improve any of the aforementioned microstructural metrics in either cortical or trabecular bone [33].

It has been demonstrated that increased fracture risk in T2DM may be exacerbated not only by significant impairments in trabecular bone microarchitecture, but also by the quality of cortical bone. According to several studies [50,53,55], cortical bone is essential for bone strength. As already indicated by the relevant parameters of micro-CT analysis associated with resistance to torsion and breaking strength (Imax/Cmax, Imin/Cmin, pMOI), we did not detect significant differences between groups D and L using mechanical testing. For most parameters, these findings are in line with previous discoveries in ZDF rats [44,50,54]. In any case, cemtirestat treatment did not affect mechanical properties of the femoral diaphysis. In our earlier study, cemtirestat administration had no beneficial effects on femoral bone strength in STZ-induced diabetic rats as well [33].

Recently, numerous ARIs have been developed as efficient therapies for a variety of chronic diabetic complications. They are thought to indirectly affect bone metabolism by reducing inflammation and oxidative stress. Epalrestat, the only ARI currently available on the market, is intended exclusively for Eastern countries to treat patients with diabetic neuropathy [56,57]. In galactose-fed rats (an animal model for studying predominantly diabetic cataract), epalrestat has been shown to decrease the transient rise in bone resorption markers and, consequently, enhance bone volume [25]. This might indicate that the development of altered bone metabolism in galactose-fed rats is closely related to the polyol pathway. According to Strother et al. [58], however, the galactosemic rat model is not very similar to the STZ-induced diabetic rat model in extraocular muscles. Furthermore, extensive divergence in several markers of oxidative stress was found between the two models. Glajchen et al. [24] evaluated the effects of another ARI sorbinil on biochemical and bone microstructural parameters in STZ-induced diabetic rats. They discovered that sorbinil had no effect on all parameters investigated. In our previous research [33], cemtirestat failed to influence all microstructural abnormalities in cortical and trabecular bone compartments of the femur linked to T1DM in an identical rat model. This suggests that the polyol pathway may not play a significant role in the pathophysiology of diabetic bone disease in STZ-induced diabetic rats. In an experimental rat model of T2DM, the impact of ARIs on the skeletal system has not been investigated yet. Therefore, our results provide initial information in this research area. However, our findings and the evidence currently available suggest that ARIs may not be very promising as therapeutics for impaired bone turnover, disturbed bone microarchitecture, and lower bone strength due to DM, in contrast to diabetic neuropathy, retinopathy, or nephropathy. According to the HIPED database (www.genecards.org [59]), AR abundance in bone is 8.5 times lower than in kidney and 28.6 times lower than in retina. These different levels of expression and biosynthesis in various tissues may lead to differences in sorbitol production in cells and ultimately explain the differential efficacy of ARI treatment in diabetic bone disease. In addition, the involvement of various glucose transporters may affect glucose uptake in different tissue types during the hyperglycemic state. In osteoblasts, glucose uptake is mediated by GLUT4 (insulin-responsive) in addition to GLUT1 and GLUT3, with GLUT4 expression being required for proper osteoblast proliferation and differentiation [60]. On the other hand, GLUT1 is a major glucose transporter of the human retina [61], and GLUT1 and GLUT3 enable glucose utilization by peripheral nerves [62]. In any case, further research is required to completely comprehend the effect of ARIs on deteriorated bone health in both T1DM and T2DM.

Although ZDF rats are considered a suitable animal model for T2DM because they allow consistent disease progression, several differences should be considered compared to humans. These rats have a strong genetic component inherited through a single gene mutation (leptin receptor mutation), unlike the complex, polygenic inheritance of human T2DM. Additionally, feeding on a high-calorie diet is recommended to induce continuous development of DM. In this animal model, hydronephrosis and ketosis may develop [63,64]. In contrast, ZDF rats exhibit renal hypertrophy, cardiac hypertrophy, impaired longitudinal bone growth, and altered bone mechanical properties with disease progression [54,65,66]. Our results demonstrating the lack of efficacy of cemtirestat in ameliorating T2DM-related skeletal damage in ZDF rats during 2-month treatment are similar to those obtained in STZ-induced diabetic rats, a model of T1DM, where 4-month administration of cemtirestat did not improve cortical and trabecular bone microarchitecture or bone strength at cemtirestat doses of 6.4 mg/kg/day and 6.8 mg/kg/day. However, in this earlier study, cemtirestat treatment reduced plasma triglyceride levels, while plasma P levels remained unchanged [33], in contrast to our current findings. From the information mentioned above it appears that different doses of cemtirestat and/or longer duration of administration could influence certain biochemical parameters in ZDF rats, which might partially affect bone health. Since, apart from our experiment, no other studies have been conducted in animal models of T2DM focusing on changes in the skeletal system after cemtirestat treatment, further research is important to clarify this issue. However, all the data and information presented in this manuscript indicate that cemtirestat (similar to other ARIs) has insufficient impact on disturbed bone health in various animal models of DM.

## Conclusions

Two months of cemtirestat treatment did not significantly affect all bone-related parameters as demonstrated by macroscopic analysis, micro-CT cortical bone analysis, and mechanical testing in ZDF rats. On the other hand, diabetic rats receiving a lower dose of cemtirestat had considerably elevated plasma P levels and those receiving a higher dose of cemtirestat showed significantly increased trabecular BV/TV and trabecular thickness. However, the lack of efficacy of cemtirestat in improving skeletal damage associated with T2DM does not justify its use in the treatment of this chronic diabetic complication.

## Supporting information

S1 Table Mean and standard deviation (SD) values for biochemical markers, macroscopic parameters, micro-CT cortical and trabecular bone metrics, and mechanical properties of femoral bones investigated in lean ZDF rats (L), obese ZDF rats (D), and those treated with cemtirestat at doses of 2.5 mg/kg/day (DT2.5), and 7.5 mg/kg/day (DT7.5) for 2 months. (PDF)
