# Supplementary material for: Insufficient impact of the aldose reductase inhibitor cemtirestat on the skeletal system in type 2 diabetic rat model
Source: PLoS One. 2025 Nov 10;20(11):e0336508. doi: 10.1371/journal.pone.0336508 (PMC12599969; doi:10.1371/journal.pone.0336508)
Supplement: S1 Table — (PDF) [file pone.0336508.s001.pdf]

Supporting information

S1 Table. Mean and standard deviation (SD) values for biochemical markers, macroscopic parameters, micro-CT cortical and trabecular bone metrics, and mechanical properties of femoral bones investigated in lean ZDF rats (L), obese ZDF rats (D), and those treated with cemtirestat at doses of 2.5 mg/kg/day (DT2.5), and 7.5 mg/kg/day (DT7.5) for 2 months.

	**L group**		**D group**		**DT2.5 group**		**DT7.5 group**	
	**Mean**	**SD**	**Mean**	**SD**	**Mean**	**SD**	**Mean**	**SD**
**Biochemical markers**								
Glucose (mM)	10.4	2.2	26.5	4.2	26.5	5.3	26.8	5.9
Insulin (µg/L)	0.23	0.01	0.52	0.08	0.53	0.08	0.52	0.07
Triglycerides (mM)	0.93	0.04	4.53	0.32	5.10	0.32	4.54	0.35
Glycated hemoglobin (%)	4.65	0.27	14.03	0.64	13.55	0.75	13.07	0.87
Cholesterol (mM)	2.31	0.04	4.30	0.14	4.14	0.12	4.49	0.23
Urea (mM)	4.61	0.20	5.74	0.29	5.68	0.20	5.54	0.23
Creatinine (µM)	33.43	0.57	23.39	0.75	22.93	0.61	23.72	0.81
ALP (U/L)	2.42	0.4	11.8	2.58	11.5	3.26	10.88	3.36
ALT (U/L)	1.19	0.15	2.16	0.32	2.29	0.58	2.29	0.68
AST (U/L)	2.03	0.72	2.37	0.72	2.63	1.14	2.26	1.03
GGT (U/L)	0.01	0	0.04	0.02	0.04	0.03	0.05	0.03
Ca (mM)	2.61	0.20	2.90	0.66	3.09	0.37	2.90	0.28
P (mM)	1.76	0.21	2.01	0.48	2.47	0.42	2.15	0.24
Mg (mM)	0.74	0.21	0.87	0.29	0.99	0.23	0.87	0.18
P1NP (ng/mL)	45.00	13.68	42.03	6.66	37.89	8.28	42.21	12.6
CTX (ng/mL)	17.10	6.80	33.30	16.06	24.21	11.52	24.66	16.47
**Macroscopic parameters**								
Body weight (g)	343	25	437	39	437	40	430	38
Femoral weight (g)	0.71	0.02	0.79	0.07	0.80	0.04	0.75	0.03
Femoral length (cm)	3.83	0.02	3.63	0.12	3.68	0.05	3.58	0.02
**Micro-CT cortical and trabecular bone metrics**								
Cortical BV/TV (%)	98.98	0.06	98.72	0.11	98.77	0.20	98.73	0.22
Cortical BMD (mg HA/ccm)	852.7	30.2	805.8	36.8	821.9	38.9	831.8	38.9
Cortical bone thickness (mm)	0.74	0.02	0.69	0.04	0.72	0.03	0.71	0.03
Cortical bone surface (mm^2)	6.90	0.90	6.64	0.87	6.90	1.37	6.73	1.39
Cortical bone area (mm^2)	6.58	0.36	6.08	0.56	6.53	0.19	6.31	0.29
pMOI (mm^4)	14.50	1.36	13.74	2.15	14.10	0.70	12.90	1.19
Imax/Cmax (mm^3)	4.20	0.30	4.02	0.49	4.12	0.16	3.85	0.29
Imin/Cmin (mm^3)	3.35	0.30	3.29	0.43	3.40	0.14	3.15	0.25
Trabecular BV/TV (%)	15.37	2.55	8.20	4.92	10.42	3.05	15.15	3.20
Trabecular BMD (mg HA/ccm)	532.1	33.2	478.8	48.0	491.3	48.5	501.7	21.5
Trabecular number (1/mm)	1.71	0.19	1.65	0.48	1.78	0.42	2.14	0.42
Trabecular thickness (mm)	0.090	0.004	0.076	0.008	0.079	0.003	0.086	0.003
Trabecular separation (mm)	0.62	0.08	0.68	0.24	0.61	0.17	0.50	0.10
Trabecular bone surface (mm^2)	76.52	8.02	61.18	28.50	60.18	15.55	80.82	13.82

Connectivity density (1/mm^3)	38.02	5.17	28.30	14.44	27.63	8.74	38.32	8.24
Structure model index	1.74	0.28	2.42	0.42	2.32	0.14	1.95	0.23
**Mechanical properties of femoral bones**								
YPL (N)	120.49	3.58	120.97	22.73	89.66	56.14	90.64	16.06
Displacement for YPL (mm)	0.31	0.08	0.30	0.06	0.23	0.13	0.24	0.02
Energy for YPL (mJ)	18.01	5.36	17.27	6.07	12.20	8.27	9.99	2.70
Stress for YPL (MPa)	104.88	27.34	101.34	16.62	84.31	54.08	76.28	16.92
ML (N)	152.44	19.27	147.18	18.32	127.91	34.28	118.18	37.09
Displacement for ML (mm)	0.43	0.05	0.40	0.10	0.36	0.10	0.34	0.10
Energy for ML (mJ)	34.68	0.36	30.70	13.40	24.53	16.30	21.98	15.45
Stress for ML (MPa)	130.29	13.95	123.36	12.45	118.15	38.87	99.77	35.82

Abbreviations: ALP - alkaline phosphatase, ALT - alanine aminotransferase, AST - aspartate aminotrasferase, GGT - gamma-glutamyl transferase, Ca - calcium, P - phosphate, Mg - magnesium, P1NP - procollagen type I N-propeptide, CTX - C-terminal telopeptide of type 1 collagen, BV/TV - bone volume fraction, BMD - volumetric bone mineral density, pMOI - polar moment of inertia, Imax/Cmax - maximum loading resistance, Imin/Cmin - minimum loading resistance, YPL - yield point load, ML - maximum load.
